# Evaluating Vertical Malaria Community Health Worker Programs as Malaria Declines: Learning From Program Evaluations in Honduras and Lao PDR

**DOI:** 10.9745/GHSP-D-20-00379

**Published:** 2021-03-15

**Authors:** Harriet G. Napier, Madeline Baird, Evelyn Wong, Eliza Walwyn-Jones, Manuel Espinoza Garcia, Lizeth Cartagena, Nontokozo Mngadi, Viengxay Vanisaveth, Viengphone Sengsavath, Phoutnalong Vilay, Kenesay Thongpiou, Theodoor Visser, Justin M. Cohen

**Affiliations:** a Clinton Health Access Initiative, Boston, MA, USA.; b Clinton Health Access Initiative, Panama City, Panama.; c Clinton Health Access Initiative, Yangon, Myanmar.; d Clinton Health Access Initiative, Gaborone, Botswana.; e Clinton Health Access Initiative, Gracias a Dios, Honduras.; f Secretary of Health, Tegucigalpa, Honduras.; g Clinton Health Access Initiative, Vientiane, Lao People's Democratic Republic.; h Center for Malariology, Parasitology, and Entomology, Ministry of Health, Ventiane, Lao People's Democratic Republic.

## Abstract

**Background:**

Across the Greater Mekong Subregion (GMS) and Central America, governments commonly employ community health workers (CHWs) to improve access to and uptake of malaria services. Many of these networks are vertical in design, organized to extend malaria-only services to those remaining communities in which malaria persists.

**Methods:**

Between 2019 and 2020, national ministries of health (MOH) and Clinton Health Access Initiative conducted mixed-methods CHW program evaluations across the GMS and Central America. Routine surveillance and programmatic data were analyzed to quantify CHW contributions to malaria elimination objectives and identify gaps and challenges. Semistructured interviews were conducted with governmental and nongovernmental stakeholders from central to community level. This article draws comparisons between the Lao People's Democratic Republic (PDR) and Honduras CHW program evaluation results to distill broader hypotheses about how vertical CHW programs might evolve as their primary mission nears its end.

**Results:**

CHWs contribute substantially to malaria case detection and surveillance, diagnosing and treating 27% of malaria cases in Lao PDR and 55% in the department of Gracias a Dios, Honduras in 2019. In the same year, malaria test positivity neared less than 1% in both countries. In 2019, 80% of CHWs in Lao PDR and 74% in Gracias a Dios, Honduras did not report a single malaria case. From inception, both programs were organized as vertical (malaria-only) CHW programs reliant upon Global Fund financing for malaria commodities, training, supervision and, where applicable, remuneration.

**Conclusions:**

Although community case management by CHWs has been highly impactful in reducing malaria cases to near zero, new challenges of acceptability and effectiveness of malaria-only service delivery, feasibility of continued vertical program management, and sustainable financing have emerged. To achieve and sustain reductions in malaria, surveillance and delivery platforms must be redesigned to encourage (and reward) care seeking based on experience of symptoms and not on a patient or caregiver's presumptive diagnosis of disease. By expanding the roles and responsibilities of currently vertical malaria CHWs, malarial interventions can be optimized and sustained. Such a shift will also position existing community-based platforms to be resilient and responsive as epidemiology of disease and community need shift.

## Abstract (precis)

Community case management by community health workers has substantially reduced malaria across the Greater Mekong Subregion and Central America. To sustain current and achieve further reductions in malaria, surveillance and delivery platforms must be redesigned to ensure their continued use by key populations.

## Teaser

**Key Findings:**

- Community health workers (CHWs) detect nearly 30% of all malaria cases in remaining hotspots in Lao PDR and more than 50% of all reported malaria cases in select regions of Honduras.
- Despite having dedicated funding, both countries face challenges supervising, equipping, and supporting CHWs.
- As malaria test positivity declines, expanding CHWs' responsibilities may encourage individuals to seek care and enable CHWs to more promptly detect and treat remaining cases.

**Key Implications:**

- National malaria programs should assess vertical CHW network design, management, and financing to identify opportunities to promote care seeking for febrile illness.
- As likelihood of malaria as the primary cause of fever decreases, providing an integrated package of services will become more critical in ensuring that patients' needs are met.
- CHWs are a major source of malaria testing, treatment, and disease intelligence. Information systems capable of tracking their performance and progress to guide remedial action and attribute impact are essential.

Resumen en español al final del artículo.

## INTRODUCTION

Partnership with community health workers (CHWs) is paramount to achieving universal health coverage and key to accelerating progress toward disease-specific objectives.^1^ Across regions seeking to eliminate malaria, the mosquito-borne disease is increasingly concentrated within remote communities, often in locations with limited access to formal health care.^2^ Governments commonly introduce community case management for malaria by CHWs to ensure adequate coverage of malaria confirmatory diagnosis, treatment, and routine surveillance in these communities.^3^ Although the benefits of engaging CHWs to effectively extend access to care are well-documented, there continues to be debate on how their engagement should be organized.^4^ Although the World Health Organization (WHO) has recommended integration of basic services at the CHW level for nearly a decade, vertical CHW programs, organized to provide singular malaria, HIV, TB, or family planning services remain common.^5^^,^^6^

Although the design and legacy of each CHW network vary, vertical malaria-focused CHW cadres across the Greater Mekong Subregion (GMS) and Central America have contributed to significant reductions in malaria in recent years. This is perhaps most apparent in the GMS, where scale-up of malaria volunteers across Cambodia, Lao People's Democratic Republic (Lao PDR), and Myanmar has been accompanied by a reported 76% decrease in regional malaria cases between 2010 and 2018.^7^ In Cambodia, CHWs conducted more than 70% of total malaria testing in 2019.^8^ Across the GMS, multiple donors—including the Global Fund, the President's Malaria Initiative (PMI), and national governments—finance management, training, and malaria commodities for more than 30,000 CHWs. In Cambodia and Lao PDR, village health volunteers (VHVs) offering preventive and promotional services have been an integral part of the peripheral health system for decades.^9^ Since 2004, the National Malaria Control Program (NMCP) in Lao PDR has selected, trained, and vertically managed community members to test and treat for malaria in endemic districts, often drawing from the existing VHV cohort.^10^ In contrast, CHW networks in Central America were established in the 1960s and are made up of thousands of vertical (malaria-only) CHWs. Across the region, these government-run cadres identify over 50% of reported malaria cases in select malaria-endemic regions of Panama and Honduras.^11^^,^^12^

In recent years, vertical malaria-focused CHW cadres across the GMS and Central America have contributed to significant reductions in malaria.

Alongside complementary interventions such as targeted insecticide-treated bed net distribution and indoor residual spraying, CHW programs in each region have successfully contributed to substantial declines in malaria. However, achieving and sustainably maintaining malaria elimination typically require different operational approaches than burden reduction (per the WHO malaria elimination framework).^13^

Because declines in malaria incidence are inherently accompanied by reduced demand for malaria-only services, many historic and successful vertical CHW programs risk losing relevance from a patient, health system, and donor perspective.^14^ As such, progress toward malaria elimination in many countries (Lao PDR and Honduras included) has plateaued in recent years.^7^ Despite heavy reliance on vertical malaria CHW platforms, there has been limited quantitative and qualitative analysis of the effectiveness of vertical service delivery at the community level and its capacity to sustain malaria gains in changing epidemiological environments. Routine evaluation of the performance, utilization, and perceived effectiveness of existing networks will thus be essential to ensure existing CHW programs are nimble and optimally positioned to help finish and sustain the task at hand. This article presents results from evaluations of vertical community-based malaria programs in Honduras and Lao PDR, countries where malaria incidence has declined by 90% and 75%, respectively, since 2015.^11^^,^^15^^–^^18^

Because declining malaria incidence leads to reduced demand for malaria-only services, many vertical CHW programs risk losing relevance.

## METHODS

### Objectives

**FIGURE 1:**
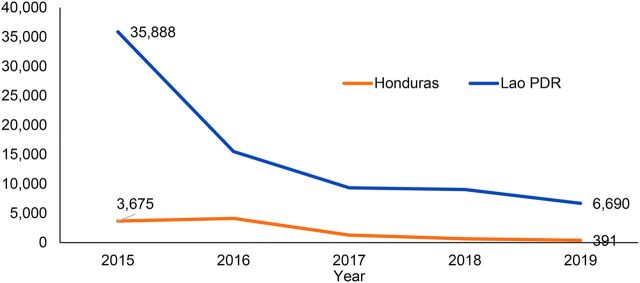
Reported National Malaria Cases in Honduras and Lao PDR, 2015–2019^11^^,^^18^ Abbreviation: PDR, People's Democratic Republic.

In 2019 and 2020, the Clinton Health Access Initiative (CHAI) collaborated with representatives from ministries of health (MOHs) in the GMS and Central America to carry out mixed-methods evaluations of government-owned malaria CHW networks. The evaluations sought to: (1) describe the demographics of the CHW cadre and contributions to malaria case management in each country; (2) document ongoing implementation of community case management programs; and (3) identify areas for network strengthening. This article presents findings from 1 country in each region to look at similarities and differences across 2 contexts: Honduras and Lao PDR. Implementation of the CHW evaluations was timed to generate recommendations for inclusion in both countries' 2020 Global Fund malaria grant applications. This article aims to share these findings and outline common challenges and needs that malaria elimination programs may encounter in environments of significantly reduced malaria incidence (Figure 1).

### Site Selection

**FIGURE 2:**
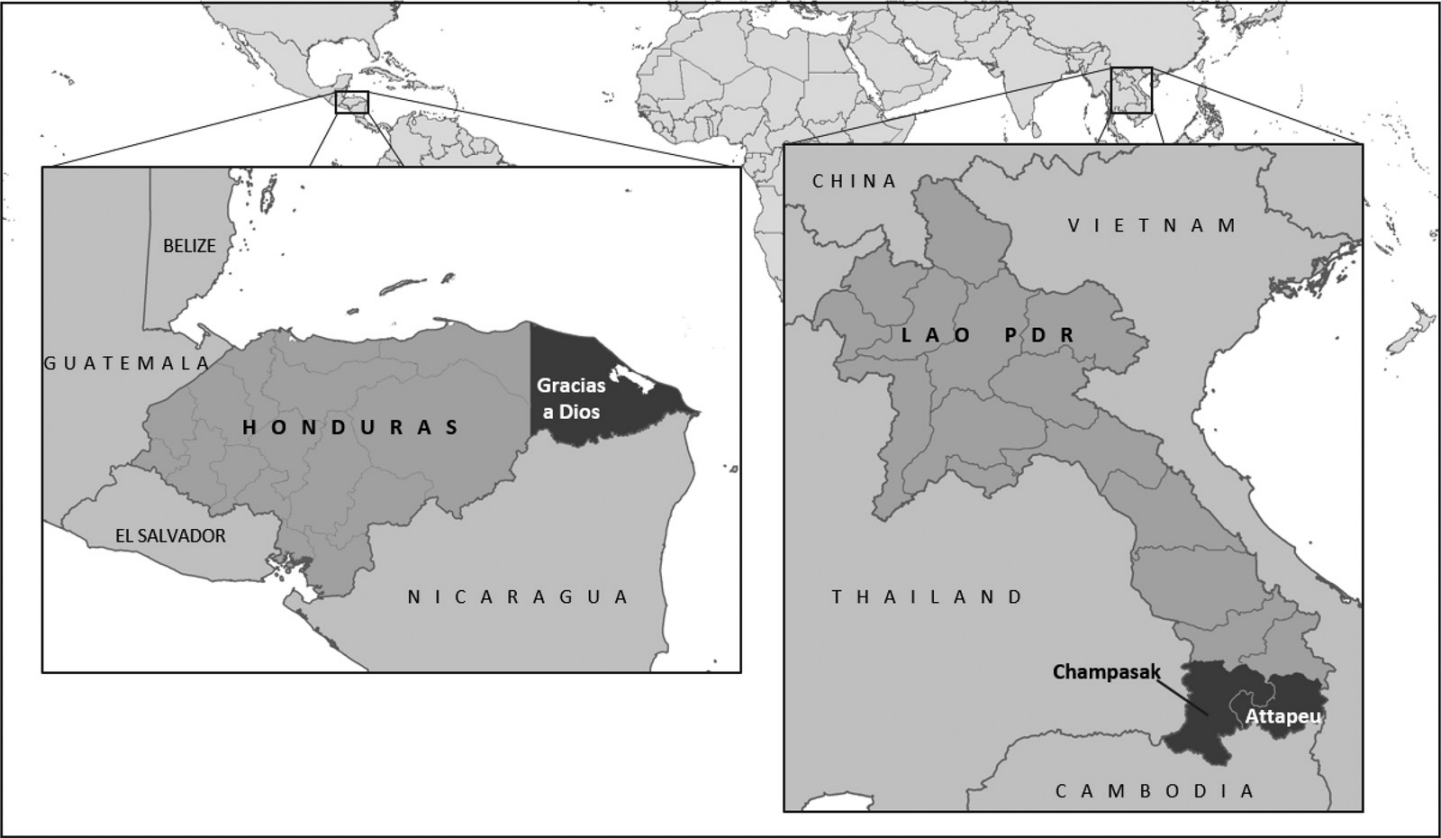
Sites in Gracias a Dios in Honduras and 2 Provinces in Lao PDR Selected for Data Collection Abbreviation: PDR, People's Democratic Republic.

The evaluations were performed in Gracias a Dios, Honduras, (reporting 60% of total national malaria cases in 2019) and Champasak and Attapeu provinces in Lao PDR (reporting 39% of total national cases in 2019). Sites for field visits and data collection in Gracias a Dios were selected to include a mix of areas with and without active malaria transmission and in consideration of population mobility, importation of malaria from neighboring countries, and CHW activity levels. Sites for field visits in Champasak and Attapeu were selected to include a mix of governmental and nongovernmental CHW program implementers, malaria burden, and CHW activity levels. Figure 2 shows the 2 subnational geographies selected for the CHW program evaluations in Honduras and Lao PDR.

### Data Collection

The program evaluations used qualitative and quantitative methods to assess program design, policy, management, and practice (Table 1). Quantitative routine program and surveillance data were reviewed to measure CHW program performance, extension of service coverage through increasing malaria case detection and appropriate adherence to case management protocols. Quantitative data sources included district health information system (DHIS2) surveillance data and programmatic data, such as supervision and training information, collected via Google and Excel databases as part of routine network monitoring and evaluation. Qualitative methods were employed to assess perceptions of the CHW programs by multiple stakeholders. In both countries, semistructured interview guides were developed in coordination with representatives from the MOH and other partner organizations and in consideration of WHO and other (i.e., CHW Assessment and Improvement Matrix toolkit) guidance on the core components of CHW systems.^19^

**TABLE 1.** Description of Qualitative and Quantitative Methods and Indicators Used in Honduras and Lao PDR Evaluations of Government-Owned Community Health Worker Networks

	Honduras	Lao PDR
**Primary outcomes**	Health system and community member perspectives on CHW network performance and relevance while providing malaria-only services as malaria incidence declines, across department	Governmental and nongovernmental perspectives on CHW performance, contribution, and program design from central to community level
**Quantitative data**		
**Data sources**	DHIS2 surveillance data from January 2017 to December 2019, and operational data from Excel databases related to supervision, training, and network management	DHIS2 surveillance data from January 2018 to December 2019, and programmatic data stored via Google Sheets and Excel databases related to CHW demographics, network stocking, training, and reporting
**Indicators or components included**	Total cases captured by CHW network Proportion of cases detected by CHWs Test positivity rate (country and study site) Total malaria tests collected across department CHW pre/post-test training scores Time between symptom onset and diagnosis^a^ CHW demographic information including average CHW length of service, sex, and age Supervisor to CHW ratio by municipality^a^	Total cases captured by CHW network Proportion of cases detected by CHWs Test positivity rate (country and CHW^a^) Total malaria tests collected across country CHW pre/post-test training scores CHW demographic information including average CHW length of service, sex, age, and education CHW reporting timeliness and completeness^a^ CHW average testing activity^a^
**Qualitative data**		
**Data sources**	40 stakeholder interviews and community focus groups, including in-depth interviews at ministry of health at central, regional, local levels (n=16); interviews with CHWs (n=19); focus groups with community members (n=39)	34 interviews, including in-depth interviews at central National Malaria Program and partners (n=8), province (n=5), district (n=5), health center (n=6), and CHW level (n=10)
**Indicators or components included**	6 elements of CHW systems included in questionnaire: (1) network management; (2) training; (3) supervision and supply chain; (4) reporting and network monitoring and evaluation; (5) health system linkage; (6) service provision and community participation	10 elements of CHW systems included in questionnaire: (1) program objectives; (2) management, leadership and governance; (3) terms of reference; (4) training; (5) payment processes; (6) supply chain; (7) supervision and performance management; (8) information management; (9) health system linkage; (10) community engagement and utilization

Abbreviations: CHW, community health worker, DHIS, district health information system; PDR, People's Democratic Republic.

a Distinct indicators reviewed according to country-specific available programmatic and surveillance data.

For both evaluations, field-based interviews were conducted with representatives from multiple partners involved in the management and implementation of the CHW program. In Honduras, field visits occurred in February 2020. Interviewees included representatives from multiple levels of the national health system, malaria program implementers, and community members. In Lao PDR, qualitative data collection occurred in May 2019. Interviewees included governmental and nongovernmental representatives from national to village level.

Interviews were conducted by CHAI and MOH staff in local indigenous or national language (Miskito or Spanish in Honduras and Lao in Lao PDR) according to stakeholder preference. In-person translation was provided by either CHAI or MOH staff, and interview details were documented throughout data collection. In Lao PDR, 2 notetakers were used where possible. In both countries, identifying participant information was not collected, and all participants provided oral informed consent before participation.

### Data Analysis

In both Honduras and Lao PDR, evaluation data were analyzed collaboratively by CHAI, MOH, and other relevant stakeholders (e.g., implementing partners). Evaluation data were originally presented as individual country CHW program reviews and incorporated into each country's malaria Global Fund application. Lao PDR's evaluation was also incorporated into the annual national malaria program review and subsequent strategic documents. These evaluations were conducted as routine MOH programmatic monitoring and evaluation activities and did not require in-country ethical approval.

Qualitative data analysis for Lao PDR and Honduras consisted of coding interviews collected on paper forms according to the 10 elements of CHW systems collected in the questionnaire. Numbers and percentages were then calculated for each theme to provide a general overview of response frequency. Exceptions were noted and additional nodes were added to the coding system as needed based on additional themes that emerged during review. In Lao PDR, themes were also compared against NMCP guidelines, manuals, and quantitative data collection to corroborate interview findings.

Quantitative data were cleaned and analyzed using DHIS2 and Microsoft Excel data sources. For both studies, proportions were calculated to describe key demographic characteristics of the CHW network and CHWs interviewed. We captured case and testing contribution data using national DHIS2 in both contexts, then extracted the data, and conducted descriptive analysis of proportions in Microsoft Excel 2016. In Lao PDR, additional data on CHW-reported malaria cases and reporting contribution were extracted from GoogleSheets for descriptive analysis in Microsoft Excel 2016. In Honduras, proportions for stock and supervision data captured through SurveyCTO platform were analyzed within data capture on GoogleSheets.

## RESULTS

The quantitative and qualitative results were extracted from evaluation reports according to 5 overarching themes that emerged across country contexts.

### CHW Demographics

Details on CHW demographics for both countries can be found in Table 2. The CHW network in Gracias a Dios, Honduras, consists of 330 trained community volunteers. The average CHW age is 41 years ranging from 18 to 75 years, with 55% of CHWs having only primary-level education and 38% having a secondary-level education. Females comprise 73% of the cadre, and CHWs have an average of 10 years' experience. According to risk strata, malaria CHWs are expected to provide services to a range of 250 to 1,000 residents. The position is commonly passed from one generation of CHW to the next without standardized recruitment or certification criteria. The volunteers do not receive financial incentives or have set working hours.

**TABLE 2.** Malaria Epidemiology, Community Health Worker System Structures, and Case Management Policies, 2019, Honduras and Lao PDR

	Honduras	Lao PDR
No. malaria cases	391	6690
Test positivity rate	0.2%	1.3%
Incidence trends	90% decline since 2015	75% decline since 2015
Malaria cases detected by CHWs	55%	27%
CHWs reporting a malaria case	26%	20%
**Study Site Details**		
Study location	Gracias a Dios department	Champasak and Attapeu provinces
Study site test positivity rate	0.6%	0.8%
National cases from study location(s)	60%	39%
CHW network size (study site/national)	330/2,900	483/1,598
**Community Health Worker System Overview**		
Services provided by cadre	Curative malaria services only without paracetamol (vertical cadre)	Curative malaria services only with paracetamol & oral rehydration salts (vertical cadre) Additional health services to communities, based on request from health center staff or recruitment by other vertical programs (e.g., TB, maternal and child health)
Case detection methods	Passive and active	Passive
Gender composition of network	73% female	81% male
Average CHW age, years	41	31–50
Minimum education level	Primary education	Primary education
CHW: population ratio	1: 250 to 1,000	1: 100 to 1,700 (94% of CHWs under 1:1000)
Date CHW cadre established	1960s Malaria Eradication Campaign	2005
CHW renumeration	US$0	US$19 monthly
Key financial and operational partners supporting CHW system	Global Fund, Global Communities	Global Fund, United Nations Office for Project Services, civil society organizations
Body in charge of network management	Coordinated across integrated Ministry of Health units	National Malaria Control Program
**National Case Management Policies for Malaria**		
	Free malaria testing and treatment in the public sector	Free malaria testing and treatment in the public sector
Testing	Rapid diagnostic test (RDT) and accompanying blood slide for confirmation with microscopy for all patients with fever, chills, headache, or profuse sweating in malaria-endemic areas	RDT for all patients with fever or 2 risk factors (e.g., travel to forest and nausea), in malaria-endemic areas
Treatment	*P. vivax* and *P. falciparum* cases: chloroquine for blood-stage infections *P. falciparum* cases: single dose primaquine *P. vivax* malaria for radical cure: either 14 or 7 days of primaquine	Artmesinin-based combination therapy for all positive cases treated *P. vivax* and mixed *P. vivax*/ *P. falciparum* cases referred to health center or hospital for G6PD testing and primaquine
Referrals	Pregnancy, breastfeeding mothers, infants under 6 months of age and suspect severe malaria cases	Pregnancy, severe cases, P.*vivax* or mixed *P. vivax/ P.falciparum* cases, patients with malaria in the past 28 days

Abbreviations: CHW, community health worker; PDR, People's Democratic Republic; RDT, rapid diagnostic test.

In Lao PDR, the CHW network consists of 1,598 trained community volunteers. Malaria CHWs are expected to provide services to a range of 100 to 1,700 residents, with 94% of CHWs serving a population under 1,000. Standard recruitment criteria exist but may not be closely followed, particularly the criterion that CHWs should be aged 40 years or younger. National data collected from 913/1,598 CHWs revealed an age range between 16 and 70 years, with 53% aged between 31 and 50 years. Male CHWs make up 81% of the network. CHWs receive US$19 monthly, split between US$12 incentive and US$7 transport payments. Most (91%) CHWs reported having more than 1 year of experience, with 43% reporting 10 or more years of service. More than half (51%) of CHWs received primary education, and the rest have either secondary level education or higher. CHWs do not have set working hours and are not expected to conduct active malaria case detection.

### Malaria Case Detection and Test Positivity

Since 2015, Honduras has reported a 90% reduction in malaria cases. In 2019, the country reported a slide positivity of 0.2%, reduced from 2.4% in 2015 (capturing both active and passive case detection efforts). Lao PDR reported a 75% reduction in malaria cases between 2015 and 2019 and a decline in malaria test positivity (from active and passive case detection) from 12.6% to 1.3%.

Both countries reported drastic reductions in malaria cases from 2015 to 2019.

In Gracias a Dios, 168 CHWs were trained and equipped in 2017 with malaria rapid diagnostic tests (RDTs), in addition to the 162 CHWs that were equipped to use RDTs in years prior. Between 2017 and 2019, regional MOH staff supported efforts to increase frequency of CHW supervision and duration of CHW training. Over this period, the proportion of cases detected by CHWs doubled, from 29% in 2017 to 55% in 2019. In Gracias a Dios, 26% of the CHW network reported a malaria case in 2019. Despite an increase in proportion of cases reported at the community level since 2017, delays in malaria diagnosis and treatment persisted, with 37.5% of CHW-detected cases diagnosed more than a week following symptom onset in 2019. Interviews with MOH representatives and community focus groups attributed this delay to patients' resistance to seek care where only malaria services were offered each time they presented with fever. They also described patient preference for self-medication with locally available fever-reducing medicines to alleviate symptoms and diminishing perceived value of visiting a CHW capable of responding only to malaria. One community member stated:

*[CHWs] provide malaria tests but do not give us pills … What purpose does it serve to come to the CHW since the test always comes back negative?* —Male community member

In Champasak and Attapeu, testing by the CHW network increased by 300% between August 2018 and August 2019 following nationwide health worker training and dissemination of guidance recommending the testing of all fevers in malaria hotspots. Over this same period, the monthly proportion of total tests conducted by CHWs increased from 11% to 17%, and CHW test positivity rates decreased from 3.4% to 0.8%. Notably, of the 971 CHWs nationally with complete data from January to April 2019, 777 (80%) did not encounter a single instance of a positive malaria test. Reports of low positivity were supported by qualitative interview data. Of the 6 CHWs interviewed in Champasak, 3 mentioned seeing no cases in the past month, and 1 CHW mentioned that he had not seen a positive case in the past 3 years.

### Management, Financing, and Performance Monitoring

In Lao PDR, the NMCP oversees management of the CHWs, working in coordination with implementing civil society organizations (CSOs). The NMCP is organized into specialized units dedicated to program management, epidemiological surveillance, case management, health education, and vector control. In Honduras, the malaria program is coordinated across integrated central level MOH units, including laboratory, epidemiology, and health service provision. The principal recipient of the Global Fund investment for Honduras, Global Communities, coordinates with the MOH and partners to support the CHW network.

In both Lao PDR and Honduras, Global Fund allocations finance most of the routine CHW trainings, supervision, and malaria commodities. The CHW evaluations identified network management challenges linked to dependence on narrow, external financing (primarily Global Fund) and bottlenecks to effective partner coordination. In Honduras, MOH stakeholders described challenges with planning trainings and activities that are largely dependent on the availability and accessibility of external financing. In Lao PDR, the evaluation identified a lack of regular CHW program planning and coordination mechanisms. Stakeholders identified that the absence of a central coordinating unit to facilitate CSO and NMCP coordination inhibited the prompt resolution of CHW operational challenges, such as delayed or disjointed incentive payments.

Network management challenges were linked to dependence on narrow, external financing and bottlenecks to effective partner coordination.

Regional or provincial level interviewees in both countries highlighted the importance of simple reporting processes and systems to effectively monitor CHW performance. In Lao PDR, CHW location and demographic data are collected at recruitment in a Google database, which, when combined with detailed CHW testing and case data on a monthly level, allow for a granular understanding of the network. Every 6 months, MOH and CSOs circulate an updated CHW location list. However, multiple interviewees described this reporting system as time-consuming, duplicative, and challenging to update with multiple information systems (i.e., DHIS2 and Google Sheets) and stringent financial reporting requirements. In Honduras, CHW data are stored within a centrally managed DHIS2 platform and regionally managed Excel databases. Interviewees described direct field visits to CHWs as the principal means for monitoring them due to challenges with timely information flow from communities, unreliable data quality, and duplicative data collection. In both Lao PDR and Honduras, stakeholder interviews highlighted operational challenges managing multiple data sources, including difficulties with routine network monitoring and accurate data on CHW program demographics, contributions, performance, and attrition.

### Supervision and Supply Chain

In Honduras, environmental control technicians (MOH staff who primarily support vector-borne disease interventions) provide monthly community-level supervision to CHWs to collect reports and restock commodities. Interviews with MOH representatives described difficulties monitoring CHW supervision and allocating sufficient resources for monthly visits. Stakeholders identified limited transport, walking distance of more than 10 hours, and a burdensome CHW to supervisor ratio (as high as 29:1) as barriers to effective supervision. Programmatic monitoring and evaluation data from visits by regional MOH staff to CHWs revealed that 18% of CHWs had a stock-out of at least 1 essential case management commodity (blood slides, malaria RDTs, or antimalarial treatments). Although interviewees described supervision improvements associated with provision of monthly transport stipends to CHW field supervisors since 2018, they emphasized the continued importance of sufficient financing for field supervisors to reliably carry out monthly CHW supervision.

In Lao PDR, provincial and district staff described that they were unable to conduct regular field-based CHW supervision due to coordination and resource gaps. Instead, monthly CHW meetings at health centers served as the primary opportunity for CHW report submission, stock replenishment, mentorship, and payment. All CHWs interviewed reported traveling to the health center the first week of each month. Strong linkages between districts, health centers, and CHWs were reported as contributing to a high average CHW reporting rate of 92% nationally. Few stock-outs of malaria commodities (artemisinin-based combination therapy, RDTs, and primaquine) were reported by CHWs. Some commodities such as gloves and first aid kits, that are neither provided nor tracked by the malaria program, were reported as inconsistently available to CHWs.

### Health System Integration

In Gracias a Dios, malaria trainings are typically managed by regional MOH staff in coordination with CHW field supervisors, with limited participation from surrounding health centers. All 7 central and regional level MOH representatives interviewed stated that additional training for CHWs in other disease areas would be beneficial, yet cautioned on overburdening the volunteer network. Two MOH representatives described the benefit of integrating tasks to enhance CHW motivation and sustain community surveillance:

*With the recent reduction in cases, it will require more training of the CHW network so that they do not become demotivated.* —MOH staff, Gracias a Dios

*The redirection of trainings [is important] so that CHWs feel motivated to continue looking [for fevers] through adding other services to the network for more holistic medical attention.* — MOH staff, Gracias a Dios

In Honduras, CHWs reported little to no involvement in broader health system activities and described challenges referring patients to surrounding facilities. CHWs reported serving their communities as their principal motivation in their role and described this to be increasingly difficult due to frequent negative malaria test results. Given CHW kits in Honduras only include malaria tests and antimalarial treatment (without paracetamol), 16 of 19 (84%) CHWs interviewed reported needing additional medicines to serve in their role, and the same proportion reported the community requesting CHW services beyond malaria diagnosis and treatment. Community members alike described the importance of altering services available through the CHW network, mentioning that malaria had ceased to be a major problem in comparison to other health problems such as general fever and access to potable water. CHWs and community members stated that an integrated package of CHW services would also enhance prompt treatment seeking. One FGD member stated:

*If the CHW had a medicine kit of more medicines, [I] would go to the CHW as soon as I felt ill.* —Male community member

Community members in Honduras said because malaria was no longer a problem compared to other health problems, CHWs should provide other services.

Community members and MOH stakeholders identified treatment for childhood diarrhea, pneumonia, TB, and dengue, as well as maternal and child health and first aid as priorities for CHW service integration. All but 1 CHW interviewed (18 of 19) affirmed their willingness to receive additional medicines and their readiness to provide health services for other diseases to their communities.

In Lao PDR, malaria training is conducted through cascade training, where central level trainers train provinces and district malaria staff, who in turn train staff at hospitals, health centers, and CHWs. Both MOH and CSO CHWs reported receiving the same training and materials, such as RDT job aids and treatment algorithms. When asked if they would benefit from additional training in qualitative interviews, half of CHWs indicated that they would benefit from more training on malaria testing, treatment, and counseling and requested additional training in other disease areas such as dengue, diarrhea, and pneumonia.

In contrast to Honduras, subnational and community interviewees in Lao PDR reported that in addition to their passive case detection responsibilities, malaria CHWs are often enlisted by their communities and nearby health centers to perform additional health activities, primarily for TB, maternal and child health, vaccinations, and health promotion. In contrast to the information in the CHW database wherein 635 of 867 (73%) of CHWs self-reported as malaria-only volunteers, most CHWs interviewed reported performing other community health roles beyond malaria and being the only CHW in their village. This overlap is likely linked to the coexistence of the VHV program in many of the communities.

Although utilization data were not collected during the interviews, a minority of CHWs reported that community members visited them whenever they had a fever, and all CHWs said the most requested service was an RDT. In addition to the standard malaria diagnostics and medicines, oral rehydration salts and paracetamol are provided to all CHWs, with some CSOs providing a supplemental first aid kit. Despite serving in additional capacities within their communities, CHWs report frequently encountering patients with signs of illness they could not treat, as described in CHW case management guidelines in Table 2.

## DISCUSSION

CHW program evaluations from Honduras and Lao PDR highlight CHWs' significant contributions to malaria case management and surveillance and describe varying interactions between CHWs and their local primary health care system. Both countries struggle with reliable field-based supervision of hard-to-reach CHWs, though Lao PDR seems to have largely resolved this bottleneck by shifting primary supervision to the health facility and compensating CHWs for their transport to and from. Strong linkage of CHWs to a comprehensive primary health care clinic may also offer an effective route for improved timeliness and completeness of reporting and commodity security. Near complete reliance on a single source of financing poses threats to both programs, as does the absence (Lao PDR) or limited use (Honduras) of existing integrated MOH coordinating mechanisms. Where the work is unpaid, the CHW cadre is predominantly female, a situation observed within other CHW program evaluations globally.^20^ Although the CHW program in Lao PDR is coordinated, financed, and managed by the NMCP as a vertical platform, the obvious overlap between CHWs and VHVs appears to have improved receptivity of CHWs and their contributions at community and health center levels despite declining rates of malaria.

Despite the CHW-VHV overlap in Lao PDR, both program evaluations describe emerging challenges these cadres face in providing malaria-only services as test positivity declines below 1%. Results from Honduras suggest that vertical service delivery in a low incidence setting may erode community trust, delay care seeking, and demotivate CHWs who find themselves unable to meet community health needs. Regardless of the involvement of Lao PDR's CHWs in other health activities, the continuation of nonmalarial activities relies heavily on external malaria financing, which allows CHWs to travel with relative ease between their communities and nearby health centers—a clear benefit for malaria and nonmalaria activities. In addition to making a formerly vertical platform more versatile, we hypothesize that broadening the purview of CHWs in these communities may have the unexpected benefit of more rapid identification of the few remaining malaria cases.

These findings echo the importance of “community embeddedness” in CHW program design and suggest that the package of services with which CHWs are equipped may differently influence patient care seeking and CHW job satisfaction.^13^^,^^21^^,^^22^ Studies conducted in low malaria prevalence contexts in Myanmar and Cambodia support these conclusions, citing a doubling in malaria testing by formerly malaria-only CHWs (Myanmar)^14^ and increases in both utilization of CHW services and CHW motivation (Cambodia)^23^ following an expansion of CHW services. We surmise that expanding CHW capacity to respond to other causes of illness in Honduras and strategically employing the overlap between CHWs and VHVs in Lao PDR may increase the odds that community members will (1) elect to seek care from the CHW; (2) be diagnosed accurately and registered as such within national disease surveillance systems; (3) receive appropriate treatment; and (4) continue to seek health services at points of care endorsed, supported, and monitored by national programs. These conclusions are consistent with the literature on the benefits of multi- versus single-disease CHW programming from effectiveness and efficiency perspectives.^24^^–^^26^

These findings echo the importance of community embeddedness in CHW program design—equipping CHWs with a certain package of services may influence patient care seeking and job satisfaction.

Successful integration will depend on government capacity to adapt national policy and absorb costs and management structures required to sustain these networks.^27^^,^^28^ As malaria epidemiology shifts, resource envelopes change, making CHW program evolution essential to ensure the continued productive interaction between CHWs, community members, and the health system.^25^ As mentioned, malaria CHW programming in both Honduras and Lao PDR relies heavily on Global Fund financing, which by rule does not fund the nonmalarial commodities (such as oral rehydration salts or amoxicillin) that come with a shift to integrated service delivery.^29^ If health systems or primary donors are incapable of covering these costs, cofinancing opportunities must be identified in the mutual interest of malaria and nonmalaria gains. Interviews with MOH officials in both Honduras and Lao PDR found a high level of receptiveness to the introduction of additional disease tasks within the CHWs' scope of work. In contrast to the highly vertical central management of the CHW network in Lao PDR, integrated CHW program management by multiple MOH departments in Honduras may offer an avenue for evolution of the longstanding malaria-only CHW network. Despite observed global encouragement by technical partners and donors, formal guidance remains limited on how health system actors can effectively transition an existing CHW program from vertical to integrated service delivery.^30^^,^^31^

Previous studies have focused largely on how integration of tasks can increase care seeking and confirmatory malaria testing at select points of care. This article adds to the literature by discussing system-wide considerations inherent to such a transition related to health system linkage, management, and financing. From a health system perspective, the investment required to collect and compile reports, perform supervision, and distribute commodities and compensation becomes less sustainable as malaria becomes a less urgent disease. From a donor perspective, the financing required for each malaria case captured increases steadily. As financial and administrative costs increase on a per malaria case basis, the marginal returns of the program in the eyes of donors, MOH officials, and the community begin to diminish. These issues have been insufficiently discussed in the malaria space.

This article discusses system-wide health system linkage, management, and financing considerations inherent to transitioning from vertical to integrated service delivery.

As malaria epidemiology shifts, resource envelopes change, necessitating that the CHW program evolve to ensure the continued interaction between CHWs, community, and the health system.

### Limitations

Several limitations may have affected the quality of the findings presented in this post-hoc comparative analysis. Interviews were not recorded, and interview notes were translated into English, introducing the possibility of translation error. In both Honduras and Lao PDR, the assessments were conducted under the auspices of the country's national malaria program in partnership with CHAI as part of routine operational evaluation and management, thus were not designed to answer specific research questions nor were they designed to be compared. Interview data from both countries come from accessible, available, and MOH-recommended communities and health centers and do not represent a random sample of stakeholder opinion.

## CONCLUSION

The substantial reductions in malaria witnessed in both Honduras and Lao PDR are not necessarily permanent.^32^ Continued vigilance against malaria by health workers and communities is required to rapidly identify imported cases, curb outbreaks, and prevent resurgence of the disease. A shift from vertical to integrated CHW programming may offer new opportunities to protect malaria progress and increase the usefulness of an existing CHW platform, but such a transition is not without its challenges. Though both vertical and integrated CHW platforms require the same basic inputs (policies, governance, financing, and data systems) and programmatic processes (recruitment, training, supervision, and compensation) as outlined by the Frontline Health project in its community health workforce performance framework, the scope and complexity of inputs and processes will change.^13^

Maintaining urgency for detecting and treating specific diseases while simultaneously sustaining integrated programs presents a significant set of operational, management, and financing uncertainties. Who within a siloed MOH will govern polyvalent programs? Will integrated programs enjoy the same dedicated financing as did their vertical predecessors? Will it be feasible to prioritize specific diseases while also fostering high comprehensive service quality and positive experience of care? How will the various elements of a CHW's scope of work be configured, operationalized, and prioritized, particularly in contexts such as Honduras where the workforce is unpaid?^30^^,^^33^ How will each country's core community health infrastructure remain simple yet dynamic, responsive, and high impact? Despite the complexity of these questions, ignoring their importance and timeliness risks atrophy of the many CHW workforces that have reduced malaria to near zero across many countries in Central America and the GMS. Even particularly successful vertical CHW programs, such as those in Honduras and Lao PDR, will need to evolve to complete their mission, sustain the gains they have achieved, and continue to advance their communities toward a healthier future.

## Translation

**En Español**

**Título:** Evaluando el programa vertical de colaboradores comunitarios mientras disminuye la incidencia de malaria: aprendiendo de evaluaciones de programas en República Democrática Popular Lao y Honduras

**Hallazgos claves**

- Los colaboradores comunitarios detectan casi un 30% de todos los casos de malaria en la República Democrática Popular Lao en los focos activos de malaria, mientras en ciertas regiones seleccionadas en Honduras, los colaboradores detectan encima de un 50% de todos los casos reportados de malaria.
- A pesar de fondos dedicados a malaria, ambos países enfrentan retos con la supervisión y apoyo brindado a sus colaboradores trabajando al nivel comunitario.
- Mientras el índice de positividad disminuye a un valor menor de un 1%, la expansión de los servicios prestados por los colaboradores puede incentivar a los miembros de la comunidad a seguir acudiendo a los servicios de salud del puesto del colaborador para malaria y posibilitar que los colaboradores puedan detectar y tratar los casos de malaria de forma oportuna.

**Conclusiones claves**

- Los programas nacionales de malaria deben evaluar el diseño, gestión y financiamiento de los programas verticales de colaboradores para poder identificar oportunidades de asegurar que los pacientes febriles sigan acudiendo a los servicios de los colaboradores.
- A medida que reduce la probabilidad de que la malaria sea la causa principal de fiebre, la provisión de un paquete de servicios integral se vuelve más importante para asegurar que los colaboradores pueden responder a las necesidades de salud de cada paciente.
- Los colaboradores sirven como un punto importante de provisión de servicios de diagnóstico, tratamiento e información de vigilancia de enfermedades al nivel comunitario. Los sistemas de información capaces de monitorear su desempeño son esenciales para guiar acciones correctivas y evaluar su impacto, tal como muestra la evidencia generada por los investigadores y actores que crean las políticas de salud.

**ABSTRACTO**

**Antecedentes:** En la Subregión del Gran Mekong (GMS) y Centroamérica, los gobiernos frecuentemente emplean a los colaboradores comunitarios para mejorar el acceso y uso de servicios de malaria. Muchas de esas redes son verticales en su diseño y están organizados para extender solamente servicios de malaria en las comunidades donde persiste la transmisión autóctona de malaria.

**Métodos:** Entre 2019 y 2020, se llevó a cabo evaluaciones de métodos mixtos de los programas de colaboradores por ministerios de salud nacionales y Clinton Health Access Initiative (CHAI) en la GMS y Centroamérica. Se analizaron los datos programáticos y del sistema de vigilancia para evaluar las contribuciones de las redes de colaboradores a los objetivos de la eliminación de malaria e identificar brechas y retos en la gestión de dichas redes. Se realizaron entrevistas semiestructuradas con representantes de unidades del gobierno y no gubernamentales del nivel central hasta el nivel comunitario. Esta investigación compara los resultados de las evaluaciones del programa de colaboradores para malaria en Laos y Honduras para proponer cómo las redes verticales de colaboradores pueden evolucionar mientras los países se acercan a su meta de eliminar malaria.

**Resultados:** Los colaboradores contribuyen de forma significativa a la detección de casos de malaria y la vigilancia comunitaria, diagnosticando y tratando un 27% de los casos de malaria en Laos y un 55% de los casos de malaria en el departamento de Gracias a Dios, Honduras. En 2019, la tasa de positividad de pruebas de malaria se acercó a una tasa menor de un 1% en ambos países. Un 80% de colaboradores en Laos y un 74% de colaboradores en Gracias a Dios, Honduras no reportaron ningún caso de malaria en 2019. Desde su comienzo, ambos programas fueron organizados como programas verticales de colaboradores que dependen del financiamiento de los fondos de Fondo Mundial para la compra de insumos, la capacitación, la supervisión y, donde es aplicable, la remuneración.

**Conclusiones:** A pesar del éxito de la estrategia de emplear a los colaboradores para proveer servicios de la gestión de casos a nivel comunitario en reducir la incidencia de malaria, surgen nuevos retos relacionados con la aceptabilidad y eficacia de los servicios únicamente para malaria, la viabilidad de la gestión continua de un programa vertical, y el financiamiento sostenible. Para lograr y sostener la reducción en la incidencia de malaria, se requiere la reestructuración de las plataformas de provisión de servicios y vigilancia comunitaria para incentivar y recompensar a los pacientes febriles que acudan a servicios de salud del puesto del colaborador. A través de la expansión de los roles y responsabilidades de los colaboradores verticales para malaria, se puede optimizar y sostener las intervenciones de malaria. Este tipo de cambio de diseño puede preparar a las plataformas comunitarias con la resiliencia y capacidad de respuesta mientras la epidemiologia de malaria y las necesidades de salud de la comunidad se van transformando.

**Peer Reviewed**

**Funding:** Clinton Health Access Initiative authors acknowledge funding support from the Bill and Melinda Gates Foundation (OPP1109772).
